# Differences in Leaf Morphological Parameters of Pear (*Pyrus communis* L.) Based on Their Susceptibility to European Pear Rust Caused by *Gymnosporangium sabinae* (Dicks.) Oerst.

**DOI:** 10.3390/plants10051024

**Published:** 2021-05-20

**Authors:** Katrīna Kārkliņa, Gunārs Lācis, Baiba Lāce

**Affiliations:** Institute of Horticulture, Graudu 1, LV-3701 Dobele, Latvia; katrina.karklina@llu.lv (K.K.); gunars.lacis@llu.lv (G.L.)

**Keywords:** European pear rust, *Gymnosporangium sabinae*, leaf anatomy

## Abstract

European pear rust is an important disease; however, the relationship between its causal pathogen *Gymnosporangium sabinae* (Dicks.) Oerst. and host *Pyrus communis* L. is poorly understood. In this study, disease severity was measured, and leaf samples were collected over three years, and their leaf water content; leaf area; leaf mass per area; and epidermis, mesophyll, and vascular tissue width and stomatal density were measured and compared between susceptible and resistant genotypes for each year. Most genotypes either showed consistent disease symptoms or showed no symptoms during the study in terms of their susceptibility. A correlation between disease severity and mesophyll tissue thickness, and stomatal density and differences between several morphological parameters were found depending on the genotype’s susceptibility. The study showed that the following pear morphological traits were stable between the years: water content, leaf mass per area, spongy mesophyll thickness, phloem thickness, and stomatal density. When selecting for breeding, we found that candidates for traits that discern susceptible genotypes from resistant were mesophyll layer width, stomatal density, epidermis width, and xylem tissue width.

## 1. Introduction

European pear rust (EPR) is an important disease found on pears and junipers, widespread where the hosts are growing together [1,2]. It is caused by *Gymnosporangium sabinae* (Dicks.) Oerst., a parasitic fungus commonly found in Europe, Asia, Africa, and North America [3]. *G. sabinae* belongs to the division *Basidiomycota* and the rust fungus order *Pucciniales*, which are characterized by being obligate biotrophs—rust fungi can only infect living host cells [4]. Rust fungi are the largest phytopathogenic fungus group; a third of all currently known *Basidiomycota* are rust fungi [5]. Studies on pear rust mainly focus on disease distribution, including discovering new infection cases in previously unaffected areas and analyzing the environmental factors contributing to fungal spore distribution and host infection. Three factors are essential in the spread of rust infection: temperature, relative humidity, and precipitation [6,7]. The infection on pear leaves is characterized by bright orange spots, where the spermagonium grows in the middle in small, dark dots [8]. These formations are harmful to the plant as they inhibit photosynthesis, increase respiration, and ultimately lead to the death of the infected organs [9].

Plant–pathogen interactions can be reflected via leaf morphology changes, which in turn are linked to leaf anatomy. Water content (WC) indicates water volume in a fresh leaf and is used as a parameter to characterize plant water status and water stress [10]. Typically, water content is explored in the context of drought stress—drought-tolerant plants not only maintain a higher leaf water content but also show higher photosynthesis rates [11]. Pathogen infection is no exception—infected leaves have lost water due to injury [12]. Leaf area (LA) is observed in tandem with leaf dry weight (DW) to measure leaf mass per area (LMA)—a parameter that also provides an understanding of the thickness and density of the leaves, which can designate sizes of cells [13] and intracellular spaces [14]. A high LMA can mean a thicker epidermis or mesophyll layer in leaves [15], contributing to leaf density. Denser leaves with less intracellular space are related to plant resistance to pathogens—tightly packed cells make it harder for a pathogen to advance into the plant’s tissue [16].

When infecting a leaf, a pathogen must face the first barrier, the cuticle—a hydrophobic layer composed of cutin and lipids [17]. The cuticle offers mechanical defense, and the hydrophobic layer makes it harder for spores to stick to the leaf [18]. In case of infection, the cuticle sends out signals to activate a plant’s systemic resistance [19], thus boosting other defense responses. Pathogen infection can cause changes in plant tissue; for example, due to rapid cell growth and division, the epidermis and mesophyll layers may increase in size [20] as a defense response to the presence of a fungal pathogen’s haustoria. On the basis of an individual plant’s resistance or susceptibility, anatomical structures can differ within a species—susceptible apple cultivars have been recorded to have a thicker epidermis and mesophyll layer than resistant ones [21], which could be related to a more severe reaction to pathogen invasion.

Plant stomata have many functions, but they can also be an indicator of pathogen resistance. Several studies have shown that susceptible cultivars have a higher stomatal density [22,23,24]. The interaction between pathogen and plant stomata is still being discussed, however. A theory could be that environmental factors that influence stomatal opening or closure could either facilitate or disrupt pathogen infection [25], meaning that stomatal density itself is not crucial in pathogen resistance.

Considering studies on *G. sabinae* and pears’ interactions are scarce, such a study would provide data specifically for the two organisms to better understand plant–pathogen interactions in a heterogeneous environment. This study aimed to identify pear morphological traits related to resistance towards EPR and evaluate the applicability of these traits in selecting resistant and susceptible genotypes.

## 2. Results

### 2.1. Evaluation of G. sabinae Severity

The first symptoms of European pear rust (EPR) were discovered on 22 May in 2018, on 27 May in 2019, and on 12 June in 2020. The highest number of trees had been infected by pear rust in 2020—12 out of 26 genotypes had visible symptoms. However, the number of trees infected was similar each year, as 10 genotypes had visible symptoms in 2018 and 11 had visible symptoms in 2019.

The average infection rate was highest in 2019; however, the differences between years were not statistically significant (*p* > 0.05). Three genotypes (2-9-2v, 5-3-1v, 5-7-1v) had four points, the highest score recorded in 2019. The highest score in 2018 was three points recorded for two genotypes—1-10-2v and 5-3-1v; the highest score in 2020 was four points for genotype 5-3-1v (Table S1).

Compared to previous greenhouse and field evaluations, genotype 1-3-2v is the only true outlier as it consistently showed no symptoms in the field in the 2018–2020 period, despite being a susceptible genotype and showing symptoms previously. On the other hand, genotypes 4-1-1v and 4-9-1v consistently showed no symptoms despite one outlier year of each—4-1-1v showed symptoms in 2018 and 4-9-1v showed symptoms in 2020; in both cases, the disease severity was low. On the basis of these results, we regarded genotypes 4-1-1v and 4-9-1v in the further analysis as susceptible genotypes when comparing means between groups, despite initially being regarded as resistant as per Table S1. The rest of the evaluated genotypes aligned with the previous data—susceptible genotypes showed symptoms throughout the evaluation period, but resistant genotypes had no symptoms.

### 2.2. Evaluation of Morphological Parameters

#### 2.2.1. Leaf Water Content

**Figure 1:**
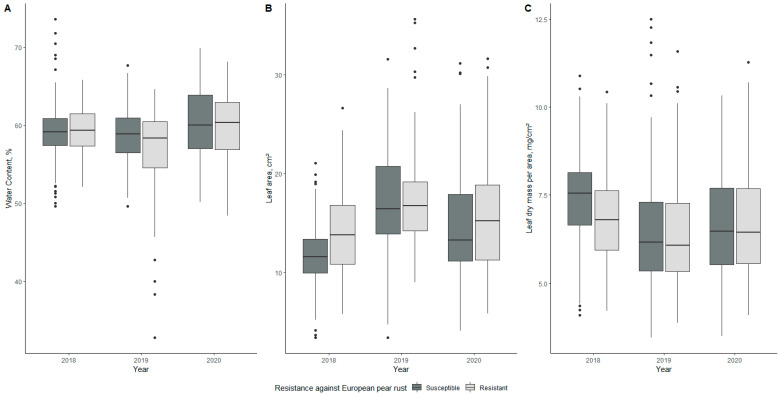
Water content (%) of resistant and susceptible pear genotypes during the evaluation period (**A**). Leaf area (cm^2^) of resistant and susceptible pear genotypes during the evaluation period (**B**). Leaf mass per area (mg/cm^2^) of resistant and susceptible pear genotypes during the three-year evaluation period (**C**). The middle line of the box plot represents the median value of measurements.

Changes in water content (WC) during the evaluation period for susceptible and resistant genotypes are shown in Figure 1A. The mean values for susceptible genotypes were 59.25%, 58.84%, and 60.39% in 2018, 2019, and 2020, respectively; mean values for resistant genotypes were 59.32%, 57.00%, and 59.59%, respectively (Table 1). The lowest WC value was found for the resistant genotype 2-4-2v in 2019, whereas the highest value was found for the susceptible genotype 1-10-2v in 2018 (Table 2). Differences in means between susceptible and resistant genotypes were significant only in 2019 (*p* < 0.05).

#### 2.2.2. Leaf Area

Differences in leaf area (LA) between susceptible and resistant genotypes are shown in Figure 1B. The mean values were 11.93, 16.79, and 14.49 cm^2^ for susceptible genotypes in 2018, 2019, and 2020, respectively; on the other hand, the mean values for resistant genotypes throughout the evaluation period were 14.16, 17.42, and 15.54 cm^2^ (Table 1). The highest value was 26.09 cm^2^ for the resistant genotype 1-1-2v in 2019, whereas the lowest value was 5.12 cm^2^ for the susceptible genotype 1-10-2v in 2018 (Table 2). Differences between susceptible and resistant genotypes were significant in 2018 (*p* < 0.001).

#### 2.2.3. Leaf Mass per Area

Figure 1C compares leaf mass per area (LMA) values of susceptible and resistant genotypes throughout the evaluation period. The mean values for susceptible genotypes were 7.42, 6.52, and 6.68 mg/cm^2^ in 2018, 2019, and 2020, respectively (Table 1), but the mean values for resistant genotypes in the same period were 6.80, 6.40, and 6.73 mg/cm^2^, respectively. The highest value was observed for the susceptible genotype 5-3-1v at 10.58 mg/cm^2^, and the lowest value was observed for the resistant genotype 4-3-1v at 4.70 mg/cm^2^ (Table 2). Statistically significant (*p* < 0.001) differences between mean LMA values for resistant and susceptible genotypes were observed in 2018.

#### 2.2.4. Leaf Epidermis

**Figure 2:**
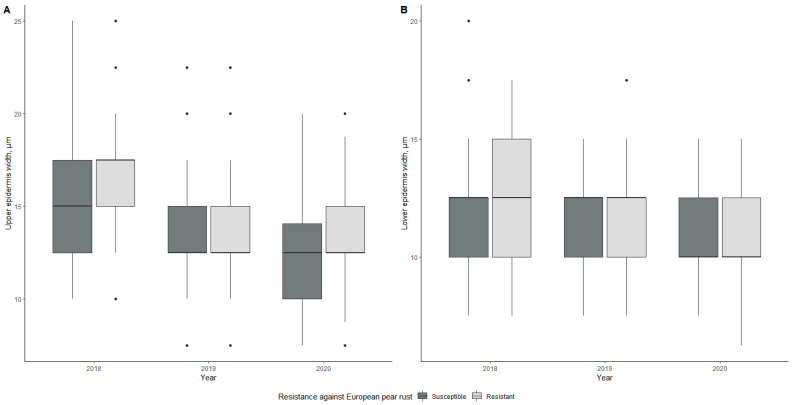
Differences in upper epidermis width (**A**) and lower epidermis width (**B**) of susceptible and resistant genotypes during the three-year evaluation period. The middle line of the box plot represents the median value of measurements.

Differences in leaf epidermis thickness are shown in Figure 2. Mean values for upper epidermis thicknesses were 15.89, 14.10, and 12.43 µm for susceptible genotypes in 2018, 2019, and 2020, respectively, and the lower epidermis thicknesses were 12.25, 11.36, and 10.67 µm, respectively, during the evaluation period (Table 1). In the case of resistant genotypes, upper epidermis values were 16.57, 13.57, and 13.44 µm and lower epidermis thickness mean values were 12.47, 11.77, and 10.42 µm in 2018, 2019, and 2020, respectively. Significant differences (*p* < 0.05) between the mean values of the upper epidermis thickness of resistant and susceptible genotypes were found in 2020.

The lowest value for upper epidermis thickness was found for the susceptible genotype 4-4-1v in 2020, with a value of 9.88 µm (Table S2). The thickest upper epidermis was observed for the resistant genotype 1-1-2v in 2018 with a thickness of 20.28 µm.

In the lower epidermis, the smallest value was found for the resistant genotype 5-2-1v in 2020 with a value of 11.13 µm, and the thickest lower epidermis was observed for the resistant genotype 2-2-2v in 2018 with a value of 16.00 µm.

#### 2.2.5. Mesophyll Tissue Thickness

**Figure 3:**
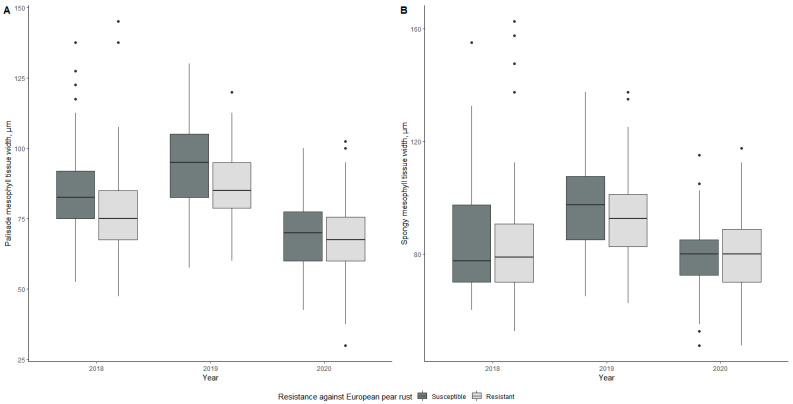
Differences in palisade mesophyll tissue width (**A**) and spongy mesophyll tissue width (**B**) of susceptible and resistant genotypes during the three-year evaluation period. The middle line of the box plot represents the median value of measurements.

Differences in mesophyll tissue thickness are shown in Figure 3. Mean palisade mesophyll thicknesses for susceptible genotypes were 84.45, 92.63, and 69.77 µm in 2018, 2019, and 2020, respectively, whereas spongy mesophyll thicknesses for susceptible genotypes were 96.76, 83.74, and 78.95 µm, respectively (Table 1). The mean palisade mesophyll thicknesses of resistant genotypes were 76.8, 86.79, and 68.60 µm in 2018, 2019, and 2020, respectively, whereas the spongy mesophyll thicknesses were 93.47, 82.40, and 80.25 µm, respectively, in the evaluation period.

The lowest mean thickness of palisade mesophyll was observed for the resistant genotype 5-4-1v in 2020, with a thickness of 55.25 µm. The lowest value for the spongy mesophyll was observed for the susceptible genotype 5-7-1v with a value of 58.93 µm (Table S2). Conversely, the highest value of palisade mesophyll thickness was observed for two years in a row (2018 and 2019) with a value of 104.50 µm—both were resistant genotypes, 2-9-2v in 2018 and 2-10-2v in 2019. However, the highest mean value for spongy mesophyll was observed in 2020 for the resistant genotype 2-2-2v with a value of 119.75 µm. Two years in a row, in 2018 and 2019, there were statistically significant differences (*p* < 0.001) between susceptible and resistant genotypes regarding palisade mesophyll tissue thicknesses. In the case of spongy mesophyll, significant (*p* < 0.05) differences were found in 2018.

Their ratio was calculated on the basis of palisade and spongy mesophyll values, and the mean values were compared. Mean values for susceptible genotypes were 1.03, 0.98, and 0.91 in 2018, 2019, and 2020, respectively, whereas, for resistant genotypes, the mean values of the mesophyll layer ratios were 0.95, 0.94, and 0.87, respectively (Table 1); however, in none of the years was the differences in means significant. The highest mesophyll layer ratio was calculated for the susceptible genotype 5-7-1v in 2020 with a value of 1.31, whereas the lowest ratio was recorded in 2020, both for susceptible and resistant genotypes—both genotypes 4-4-1v and 2-2-2v had palisade/spongy mesophyll ratios of 0.74 (Table S2).

#### 2.2.6. Vascular Tissue—Xylem and Phloem

**Figure 4:**
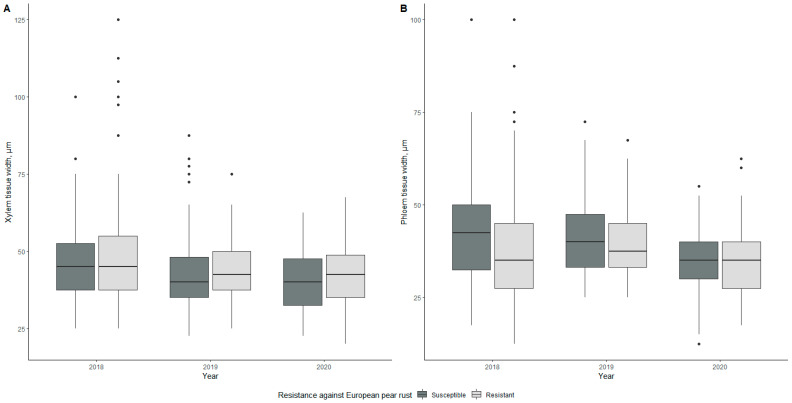
Differences in xylem tissue width (**A**) and phloem tissue width (**B**) of susceptible and resistant genotypes during the three-year evaluation period. The middle line of the box plot represents the median value of measurements.

Differences in vascular tissue thicknesses are shown in Figure 4. Throughout the three-year evaluation period, the mean values of xylem thickness for susceptible genotypes were 47.22, 42.60, and 40.29 µm in 2018, 2019, and 2020, respectively, whereas the mean values for phloem thickness were 42.78, 41.27, and 34.92 µm, respectively (Table 1). Mean values for resistant genotypes were as follows—47.98, 44.23, 42.55 µm for xylem in 2018, 2019, and 2020, respectively, and 37.72, 40.16, and 35.08 µm, respectively, for phloem.

The lowest xylem thickness value was recorded in 2020 for the susceptible genotype 5-3-1v with 29.17 µm, whereas the lowest phloem thickness was recorded in 2018 for the resistant genotype 5-5-1v with a value of 21.00 µm. On the other hand, both the highest xylem and phloem tissue thickness mean values were observed for the resistant genotype 1-1-2v in 2018 at 85.00 µm and 63.75 µm, respectively (Table S2). Significant differences (*p* < 0.001) between susceptible and resistant genotypes were observed in 2019 in the case of phloem.

#### 2.2.7. Stomatal Density

The mean values of stomatal density between 2018 and 2020 were 126, 113, and 109 stomata per cm^2^ for susceptible genotypes in 2018, 2019, and 2020, respectively, and 126, 107, and 106 stomata per cm^2^ for resistant genotypes, respectively (Table 1). The lowest stomatal density was recorded in 2020 for the susceptible genotype 1-3-2v with a value of 72 stomata per cm^2^. On the other hand, the susceptible genotype 1-10-2v had the highest stomatal density with 166 stomata per cm^2^; this value was recorded in 2019 (Table S2). No significant differences were found between susceptible and resistant genotypes.

#### 2.2.8. Correlation Analysis

Correlation analysis showed a statistically significant correlation between infection severity and mesophyll tissue thickness and stomatal density for both susceptible and resistant genotypes. Stomatal density was positively correlated with infection severity in both cases (susceptible: *r* = 0.22; *p* < 0.001; resistant: *r* = 0.17; *p* < 0.001); however, mesophyll thickness was negatively correlated for resistant genotypes (palisade mesophyll: *r* = −0.10; *p* < 0.05; spongy mesophyll: *r* = −0.11; *p* < 0.05) and positively correlated for susceptible genotypes (palisade mesophyll: *r* = 0.14; *p* < 0.05; spongy mesophyll: *r* = 0.18; *p* < 0.001).

Susceptible genotypes also had a significant correlation between leaf area (*r* = 0.23; *p* < 0.001), xylem tissue (*r* = −0.23; *p* < 0.001), and infection severity—positive in leaf area, negative in xylem thickness.

#### 2.2.9. PCA Analysis

**Figure 5:**
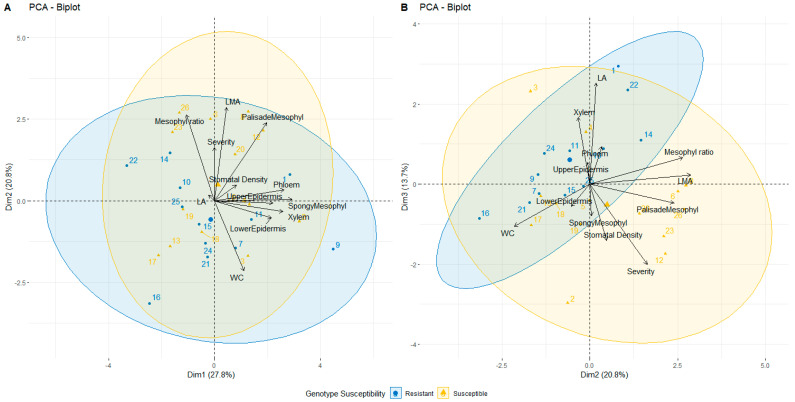
PCA analysis of the studied 26 pear genotypes and morphological traits, dimensions 1 and 2 (**A**), dimension 2 and 3 (**B**). 95% confidence concentration ellipses are drawn around the groups of resistant and susceptible genotypes.

PCA analysis was performed to discern the grouping between genotypes and the assessed traits (Figure 5); the first two dimensions explained 49% of the data variation (Figure 5A), whereas visualization of dimensions two and three (Figure 5B) showed the correlation between disease severity, mesophyll tissue, and stomatal density, which was also discovered by correlation analysis. The two dimensions also showed a more explicit grouping of genotypes and morphological parameters—resistant genotypes were grouped with epidermis and vascular tissue, whereas susceptible genotypes were grouped with mesophyll tissue and stomatal density.

## 3. Discussion

### 3.1. Evaluation of G. sabinae Disease Severity

Weather conditions affect the development of *G. sabinae* spores [26], which affects EPR severity. According to the data provided by the Latvian Environment, Geology and Meteorology Center (LEGMC) [27], the spring of 2018 was characterized with heat records—the average temperature in Dobele was 1.5 °C above the seasonal norm, and precipitation was 17% below the norm. Similar weather abnormalities were recorded in 2019 as well—the temperature was 1.6 °C above the seasonal norm, and precipitation was 46% below the norm. In contrast, in 2020, the spring temperature was 0.1 °C above the norm, but the precipitation was 35% below the norm. Previous data have shown that the period between April and May is the most important for EPR infection when basidiospores spread and potentially infect pears [28].

In April, the year with the highest monthly precipitation sum was 2018 (Figure S1), whereas in May, the year 2020 had the highest precipitation in the three years. On the other hand, the highest temperature in the three-year period was recorded in April and May of 2018.

Significant differences were found between the mean precipitation in April 2018, April 2019, and April 2020 and between the values in May 2018 and May 2019. In mean temperature values, significant differences were found between April 2018 and 2020 and between all three years in May.

Despite the amount of precipitation being similar in May 2019 and 2020, the average disease severity was lower in 2020 than in 2019 and closer to the values of 2018. This could be explained by the temperature, which is an essential factor in the EPR distribution—the temperature in May 2020 was lower than both May 2018 and 2019, which could have played a role in the development of the disease, as unfavorable conditions would have resulted in lower disease severity at the time of evaluation in July.

When comparing the data of EPR infection in the greenhouse to field data (Table S1), we found that three outliers emerged—a susceptible genotype showed no symptoms and two genotypes previously regarded as resistant showed disease symptoms during the evaluation period. Perhaps the changes in reaction to infection for these two genotypes could be explained by the nature of the pathogen—the isolates used in the greenhouse could be different from the fungus in the field. However, since the fungus itself was not assessed, further studies on the specific genotypes should be performed. The other genotypes showed stability in terms of resistance, either by not having symptoms during the 2018–2020 period or having varying degrees of symptoms in the case of susceptible genotypes, showing how a variable environment affects disease severity.

To date, in the case of EPR, there has not been any observed complete pear resistance to the pathogen, only variability of genotype severity [29,30]. Therefore, it has not been possible to identify possible plant defense mechanisms, traits that characterize it, to perform targeted breeding of resistant genotypes. Our study identified 12 genotypes that did not show any EPR symptoms in a two-year greenhouse trial with artificial inoculation [31] and in-field evaluation for four consecutive years. Although further evaluation is needed, these genotypes are considered resistant and can be used to study pear resistance to EPR and as potential donors of resistance.

### 3.2. Leaf Morphology

When considering all morphological parameters, we found that two patterns emerged—one of a steady decline over three years and one of fluctuating values, with the year 2019 standing out for several parameters. In the case of leaf water content, leaf area, and leaf mass per area, fluctuating values were observed in all cases. Palisade, spongy mesophyll, xylem, and phloem tissue showed a fluctuating pattern when it came to anatomy. The rest of the values had declining values during the study period.

It is important to note that when comparing changes in the morphology of susceptible and resistant genotypes, there was only one instance where a parameter was stable between all three years (meaning no significant differences between means)—that was the case of the xylem tissue thickness for resistant genotypes. In the case of all other parameters, there was either case where significant differences were observed between all three years or at least one case where the mean values between two years were significantly different, and it was not always the 2019 value that was the outlier.

As is expected from experiments performed in the field, the environment can play a considerable role in affecting the changes in the studied parameters. The amount of precipitation during the growing season can affect a plant in many ways—water stress has been correlated with an increase in stomatal density [32]. Another effect of the environment, namely, precipitation, is that it can also decrease plant biomass by 40% [33], affecting parameters such as leaf weight or area. On the other hand, it has also been shown that the leaf area is affected by genes and the environment [34], with the genotype playing a significant role in leaf morphology changes [35]. Leaf area reached its highest value in 2019. On the other hand, water content reached its highest value in 2020, while stomatal density reached its lowest value; thus, the environmental effect of precipitation, especially in May, throughout the years is shown in the current study results. The traits that showed relative stability throughout the years were water content, leaf mass per area, spongy mesophyll tissue thickness, xylem and phloem tissue thicknesses, and stomatal density.

The PCA analysis revealed morphological parameters that described susceptible and resistant genotypes (Figure 5). Resistant genotypes grouped with the traits upper and lower epidermis, as well as xylem. On the other hand, susceptible genotypes grouped with palisade and spongy mesophyll and stomatal density, and these parameters had a significant correlation with disease severity, despite the correlation coefficient itself being low. The low coefficient could have been due to the different factors affecting data gathered in the field; thus, a strong correlation was not shown, despite being statistically significant. Perhaps the low disease severity was what affected the correlation in general.

Different studies have concluded that pathogen resistance and thicker, denser mesophyll tissue were linked together [16,36,37]. The study’s data focused on the measured tissue layers’ size but not on individual cell sizes and density. The resistant genotypes having an overall smaller mesophyll layer width could mean that the cells are more densely packed; thus, further studies could be performed, analyzing the mesophyll closer. Another thing to note is that mesophyll thickness fluctuated similarly to the disease severity, which peaked in 2019, further showing a link between possible pathogen resistance and mesophyll tissue.

Some pathogens, for example, *Candidatus liberibacter asiaticus*, colonize host vascular tissue to acquire nutrients [38]. That means specific mechanisms are needed to protect the host; plants that have xylem-specific pathogens form special cellular structures, tyloses, as defense mechanisms [39], and phloem-specific pathogens cause the plants to limit transport through pores to stop the spread of the pathogen [40]. The study’s data showed xylem tissue being thicker for resistant genotypes and phloem tissue being thicker for susceptible genotypes. Further studies should be conducted, looking into possible resistance mechanisms as previously described to understand whether *G. sabinae* has mechanisms to ensure mechanical defense against pathogens in the vascular tissue.

Stomata can be an entry point for pathogen infection—the basidiospores of fungi grow haustoria to access the plant through many means, stomata being one of them [41]. Stomatal density can be vital for infection progress—more stomata per cm^2^ would mean more possible entryways for haustoria. Studies show that susceptible genotypes tend to have a higher stomatal density [23,24], shown in the current study’s data, even if by a small margin. When the data of different studies regarding plant–pathogen relations are compared, it is essential to note the feeding strategies of pathogens, as the results acquired in the current study might not always line up with what studies of other pathogens describe. For example, *Taphrina deformans* is a facultative biotroph [42], *Venturia inequalis* is a hemibiotroph [43], and *Xanthomonas campestris* pv. *mori* belongs to an order of necrotrophic fungi [44], but *G. sabinae* and *Phyllactinia corylea* [23] are obligate biotrophs, which, unlike the previously mentioned organisms, can only infect living cells; thus, the pathogen must elicit weak defense responses. This could mean that the differences between resistant and susceptible genotypes might not be as severe as in other plants, explaining why the differences between susceptible and resistant genotypes are not always significant.

On the basis of the correlation analysis and data shown by PCA, we could use the following traits to discern resistant and susceptible genotypes: leaf epidermis width, mesophyll tissue width, xylem tissue width, and stomatal density.

## 4. Materials and Methods

### 4.1. Site of Experiment and Plant Material

The research was performed at the Institute of Horticulture (LatHort) in Dobele (56°36′39″ N; 23°17′50″ E), Latvia. Data were collected in July 2018, 2019, and 2020 from 26 genotypes chosen to evaluate disease severity and morphological and anatomical data (one tree per genotype, Table S1). The genotypes studied were open-pollinated seedlings of *Pyrus communis* L. (local landrace “Kazraušu bumbiere”), usually chosen as rootstocks for popular pear cultivars. The susceptibility and resistance of these genotypes were assessed previously in greenhouse conditions for two consecutive years and one year in the field (Table S1). According to this initial information, we selected a set of genotypes (14 resistant and 12 susceptible). The genotypes with EPR symptoms were marked with “1”, whereas genotypes without symptoms were marked with “0”. During the three-year (2018 to 2020) evaluation period in the field, two genotypes previously counted as resistant (4-1-1v and 4-9-1v) each showed EPR symptoms in one of the evaluation years (Table S1); thus, in further data analysis, these genotypes were considered as susceptible, meaning that, overall, 12 resistant and 14 susceptible genotypes were analyzed. A genotype was considered resistant only if it had no symptoms in the greenhouse and all field trials.

### 4.2. Evaluation of G. sabinae Infection Severity

Evaluation of *G. sabinae* infection severity was performed for the whole tree once in the growing season, in July, when the infection’s development had reached its midpoint. According to the Horsfall–Barratt scale, we performed the infection’s evaluation [45], which is a 12-point scale where 1 point indicates 0% infection and 12 points indicates 100% infection. The presence of *G. sabinae* was confirmed on the basis of morphological observation of shape, color, and location of aecial horns.

### 4.3. Measurement of Morphological Parameters

Samples of leaves were taken from 26 genotypes (Table S1). Each sample consisted of 10 leaves per tree, picked randomly from around the tree. After collection, the petioles of the leaves were cut. Morphological parameters were assessed immediately after sample collection. Assessment of leaf morphological traits was performed by adapting the methods outlined by [10]. Leaf area was measured with ImageJ [46]—sampled leaves were scanned, and the image was analyzed in the program to determine the area of each sampled leaf in cm^2^. After scanning, leaf samples were dried with *Kern MLS 50-3* scales in +120 °C temperature to gather data on leaf fresh and dry mass and water content. Dry weight and leaf area were used to calculate leaf dry mass per area.

**Figure 6:**
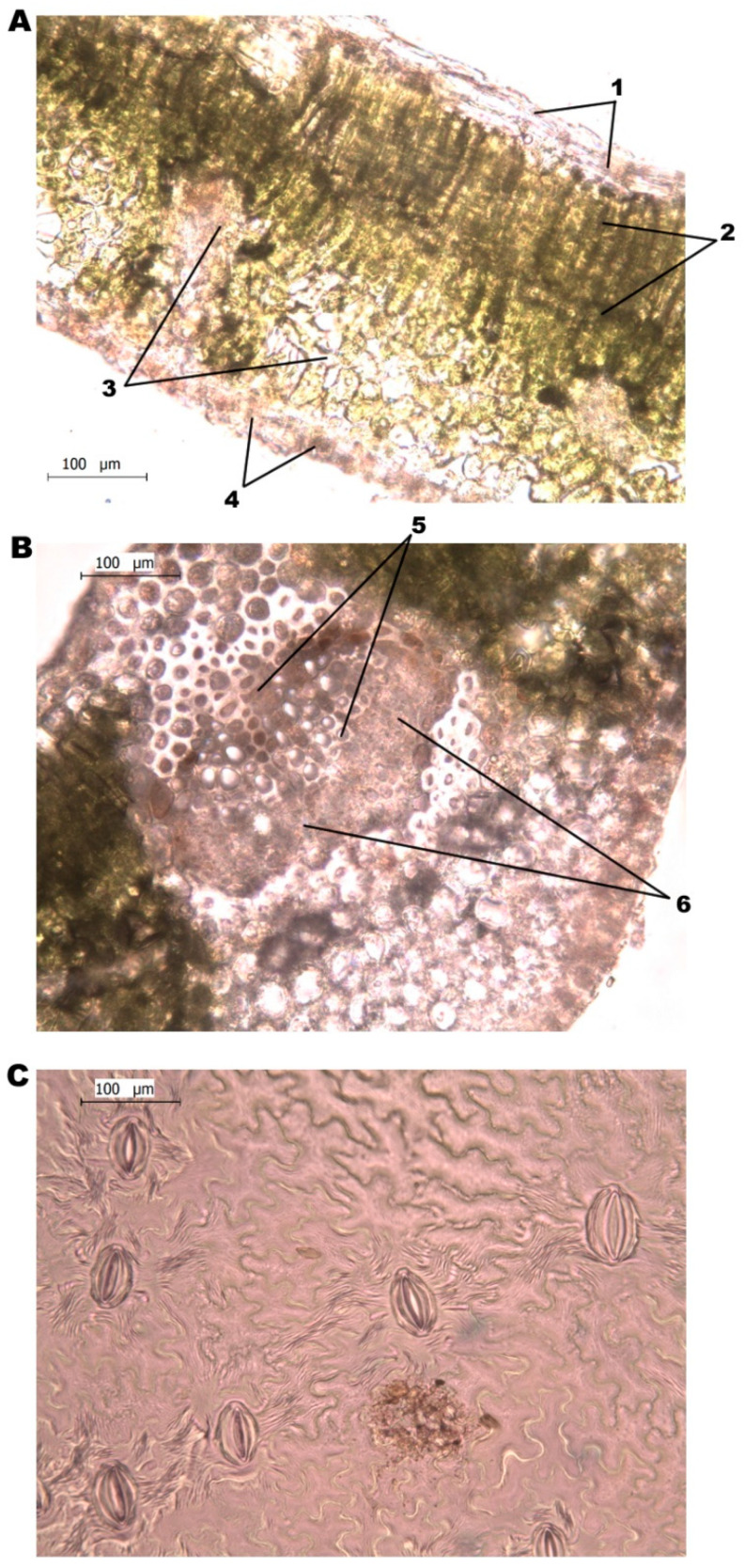
Light microscope image of leaf cross-section and leaf stomata impression (magnification 400×). Cross-section of pear leaf blade (**A**), the cross-section of pear leaf midsection (**B**), the impression of leaf stomata (**C**) (1—upper epidermis, 2—palisade mesophyll, 3—spongy mesophyll, 4—lower epidermis, 5—xylem tissue, 6—phloem tissue).

Two sets of leaf samples were taken to measure leaf epidermis, mesophyll, vascular tissue, and stomatal density—10 leaves were taken for each set the same as sampling for morphological parameters. One set of samples was used to measure stomatal density—each leaf was coated with clear nail polish on the underside. After the nail polish dried, it was removed with a strip of adhesive tape and taped to slides. Stomata were counted using a *Leica Microsystems DM/LS 020-518.500* microscope at 400× magnification. Stomata were counted in the entirety of the field of vision (FOV), including stomata, which was at least half-visible in the FOV. Cross-section cuts of the leaves were made for the rest of the samples—each sample was a fresh cut and put on a slide and observed at 400× magnification. The width (µm) of the tissue of the upper, lower epidermis, spongy and palisade mesophyll, xylem, and phloem were measured for each sample (Figure 6). The palisade and spongy mesophyll thickness measurements were used to calculate the ratio between the two parameters.

### 4.4. Statistical Analysis

Data were analyzed using R version 4.0.2. [47], and a two-tailed *t*-test or Wilcoxon signed-rank test was used to compare mean values within one year on the basis of infection or susceptibility parameters. Genotypes were grouped into susceptible and resistant on the basis of the data provided in Table S1. A Kruskal–Wallis test was performed to compare mean values between all three years, and a Spearman correlation test was performed to assess the relationship between infection severity in the field and the studied morphological parameters. Principal component analysis (PCA) was performed on the dataset to test genotypes’ grouping on the basis of susceptibility, visualizing the correlation between the selected morphological parameters.

## 5. Conclusions

The EPR severity evaluation identified 12 resistant genotypes, indicating the availability of natural resistance in pears and thus opening up the possibility to study the mechanisms of resistance in order to identify their characteristics and breed EPR-resistant cultivars. Pear morphological traits that showed stability between years were water content, leaf mass per area, spongy mesophyll tissue thickness, xylem and phloem tissue thicknesses, and stomatal density. When looking into selecting traits for resistance, mesophyll layer width (palisade and spongy mesophyll), as well as stomatal density, seem to be the best candidates for discerning susceptible genotypes. In contrast, epidermis width and xylem tissue width were defining parameters for resistant genotypes. Further studies examining the chosen parameters need to be conducted before strong statements can be made regarding their applicability in selection.

## Figures and Tables

**Table 1:** Morphological parameters of resistant and susceptible genotypes throughout the three-year study period.

Trait	Mean Values for the Morphological Parameters by Year of Measurement					
	2018		2019		2020	
	Resistant	Susceptible	Resistant	Susceptible	Resistant	Susceptible
Leaf water content (WC), %	59.32 ± 2.15	59.25 ± 3.57	57.00 ± 3.56	58.84 ± 2.41	59.59 ± 3.54	60.39 ± 2.84
Leaf area (LA), cm^2^	14.16 ± 3.66	11.93 ± 2.85	17.42 ± 3.41	16.79 ± 4.49	15.54 ± 4.48	14.49 ± 4.59
Leaf dry mass per area (LDMA), mg/cm^2^	6.80 ± 0.84	7.42 ± 1.04	6.40 ± 1.19	6.52 ± 1.47	6.73 ± 1.35	6.68 ± 1.25
Upper epidermis, µm	16.57 ± 2.10	15.89 ± 2.29	13.57 ± 1.35	14.10 ± 1.56	13.44 ± 1.50	12.43 ± 1.27
Lower epidermis, µm	12.47 ± 1.85	12.25 ± 1.31	11.77 ± 1.17	11.36 ± 0.89	10.42 ± 0.72	10.67 ± 0.63
Palisade mesophyll, µm	76.88 ± 6.08	84.45 ± 8.16	86.79 ± 7.61	92.63 ± 9.86	68.60 ± 7.66	69.77 ± 6.73
Spongy mesophyll, µm	93.47 ± 8.96	96.76 ± 8.46	82.40 ± 12.58	83.74 ± 13.03	80.25 ± 8.35	78.95 ± 7.40
Palisade/spongy mesophyll ratio	0.95 ± 0.08	1.03 ± 0.08	0.94 ± 0.07	0.98 ± 0.11	0.87 ± 0.10	0.91 ± 0.10
Xylem, µm	47.98 ± 13.91	47.22 ± 10.21	44.23 ± 6.42	42.60 ± 7.57	42.55 ± 5.83	40.29 ± 5.50
Phloem, µm	37.72 ± 11.12	42.78 ± 9.13	40.16 ± 5.38	41.27 ± 5.39	35.08 ± 5.60	34.92 ± 3.85
Stomata density, stomata/mm^2^	126 ± 17.06	126 ± 22.35	107 ± 17.40	113 ± 23.22	106 ± 17.09	109 ± 20.69

**Table 2:** Water content (WC, %), leaf area (LA, cm^2^), and leaf mass per area (LMA, mg/cm^2^) for all evaluated genotypes during the three-year study period.

	Water Content			Leaf Area			Leaf Mass Per Area		
	Year								
	2018	2019	2020	2018	2019	2020	2018	2019	2020
1-3-2v	60.94	60.35	62.56	12.62	25.11	13.46	5.76	5.57	5.20
1-5-2v	60.73	57.78	57.80	16.86	17.50	20.75	6.99	6.75	6.39
1-6-2v	61.40	56.01	64.80	16.28	20.19	11.22	7.03	6.66	5.77
1-7-2v	59.74	54.66	55.99	14.65	19.92	11.82	6.74	7.75	8.17
1-10-2v	68.71	62.85	59.46	5.12	5.59	5.13	5.50	5.15	7.46
2-9-2v	58.46	54.74	55.52	10.78	18.34	15.02	8.05	5.04	8.81
2-10-2v	58.74	58.35	58.48	11.55	17.21	12.73	7.96	7.72	8.97
4-1-1v	56.48	58.68	61.17	13.84	16.32	16.92	7.20	5.42	5.93
4-4-1v	58.30	62.49	62.27	11.80	15.16	16.56	7.37	5.50	5.75
4-7-1v	61.05	59.42	62.93	11.92	22.19	16.41	7.22	5.61	6.44
4-8-1v	60.14	60.32	65.10	9.45	14.16	10.27	7.92	5.93	4.96
4-9-1v	54.71	58.42	60.08	11.38	14.63	10.11	9.74	5.69	7.44
5-3-1v	57.26	60.67	60.09	10.67	16.35	22.69	8.27	10.58	6.59
5-7-1v	52.87	58.99	59.13	10.10	12.38	19.81	8.18	7.89	5.63
1-1-2v	61.95	56.43	60.33	19.75	26.09	20.74	5.69	8.88	8.11
2-1-2v	60.68	57.17	58.52	9.12	15.19	10.11	6.61	6.34	7.21
2-2-2v	61.13	59.06	62.94	15.70	19.43	13.41	8.29	5.34	6.48
2-4-2v	60.10	47.75	57.44	14.32	16.04	15.30	6.44	6.60	6.46
2-8-2v	63.01	52.35	61.86	20.23	17.09	16.63	6.03	6.54	7.45
4-2-1v	57.43	59.10	60.41	14.23	13.25	11.75	8.18	5.77	6.57
4-3-1v	59.36	60.22	62.77	14.17	16.39	15.26	5.65	4.70	5.42
4-10-1v	56.17	55.55	52.14	11.45	15.79	15.14	7.00	5.75	9.69
5-1-1v	59.42	59.78	63.77	10.44	16.97	12.83	7.66	5.37	4.84
5-2-1v	56.33	57.30	54.66	16.37	21.35	24.30	6.89	5.70	7.82
5-4-1v	56.74	60.62	63.22	15.95	18.04	21.84	6.88	7.96	5.03
5-5-1v	59.52	58.67	57.00	8.16	13.40	9.22	6.35	7.82	5.71

## Data Availability

The data presented in this study are available within the article and its Supplementary Materials.

## Supplementary Materials

The following are available online at https://www.mdpi.com/article/10.3390/plants10051024/s1, Table S1: List of genotypes that were sampled for assessment of morphological traits and the evaluation of European pear rust disease severity in the study period. Table S2: Mean values of leaf upper and lower epidermis thickness (µm), palisade and spongy mesophyll tissue thicknesses (µm), palisade/spongy mesophyll ratio, xylem and phloem tissue thicknesses (µm), and stomatal density (stomata/cm^2^) of all sampled genotypes during the three-year evaluation period. Figure S1: Mean temperature in the vegetation period throughout the three-year evaluation period (A) and the sum of precipitation (mm) during the three-year evaluation (B).
