# Supplementary material for: Differences in Leaf Morphological Parameters of Pear (Pyrus communis L.) Based on Their Susceptibility to European Pear Rust Caused by Gymnosporangium sabinae (Dicks.) Oerst
Source: Plants (Basel). 2021 May 20;10(5):1024. doi: 10.3390/plants10051024 (PMC8161175; doi:10.3390/plants10051024)
Supplement: Supplementary file 1 [file plants-10-01024-s001.zip › plants-1199944-SI.pdf]

Supplementary Materials

Differences in Leaf Morphological Parameters of Pear (*Pyrus communis* L.) Based on Their Susceptibility to European Pear Rust Caused by *Gymnosporangium sabinae* (Dicks.) Oerst.

Katrīna Kārkliņa [1], Gunārs Lācis [1] and Baiba Lāce [1,*]

[1] Institute of Horticulture, Graudu 1, Dobele, LV-3701, Latvia
[*] Correspondence: baiba.lace@llu.lv

Table S1. List of genotypes sampled for the assessment of morphological traits and the evaluation of European pear rust disease severity in the study period.

Genotype	Susceptibility	European pear rust symptoms* (year of evaluation)			European pear rust severity** (year of evaluation)			
		2014 (greenhouse)	2015 (greenhouse)	2016 (field)	2018	2019	2020	Average
1-1-2v	Resistant	0	0	0	1	1	1	1.0
1-3-2v	Susceptible	1	1	1	1	1	1	1.0
1-5-2v	Susceptible	1	1	1	2	2	2	2.0
1-6-2v	Susceptible	1	1	1	2	2	2	2.0
1-7-2v	Susceptible	1	1	1	1	3	2	2.0
1-10-2v	Susceptible	1	1	1	3	3	2	2.7
2-1-2v	Resistant	0	0	0	1	1	1	1.0
2-2-2v	Resistant	0	0	0	1	1	1	1.0
2-4-2v	Resistant	0	0	0	1	1	1	1.0
2-8-2v	Resistant	0	0	0	1	1	1	1.0
2-9-2v	Susceptible	1	1	1	2	4	2	2.7
2-10-2v	Susceptible	1	1	1	2	2	2	2.0
4-1-1v	Resistant	0	0	0	2	1	1	1.3
4-2-1v	Resistant	0	0	0	1	1	1	1.0
4-3-1v	Resistant	0	0	0	1	1	1	1.0
4-4-1v	Susceptible	1	1	1	2	3	2	2.3
4-7-1v	Susceptible	1	1	1	2	3	2	2.3
4-8-1v	Susceptible	1	1	1	1	2	2	1.7
4-9-1v	Resistant	0	0	0	1	1	2	1.3
4-10-1v	Resistant	0	0	0	1	1	1	1.0
5-1-1v	Resistant	0	0	0	1	1	1	1.0
5-2-1v	Resistant	0	0	0	1	1	1	1.0
5-3-1v	Susceptible	1	1	1	3	4	4	3.7
5-4-1v	Resistant	0	0	0	1	1	1	1.0
5-5-1v	Resistant	0	0	0	1	1	1	1.0
5-7-1v	Susceptible	1	1	1	2	4	3	3.0

* (yes/no) – in the years 2014-2016, EPR was evaluated based on the tree showing symptoms, 1 meaning that the tree showed symptoms, 0 being no observed symptoms. The first two years were greenhouse studies, and the year 2016 was done in the field.
**In the years 2018-2020, disease severity was evaluated in the field based on the Horsfall-Barratt scale [40] for the whole tree; a score of 1 meant the tree had 0% infection the scale went up to 12 points meaning 100% infected tree.

Table S2. Mean values of leaf upper and lower epidermis thickness (μm), palisade and spongy mesophyll tissue thickness (μm), palisade/spongy mesophyll ratio, xylem and phloem tissue thickness (μm) and stomatal density (stomata/cm²) of all sampled genotypes during the three-year evaluation period.

	Upper Epidermis			Lower epidermis			Palisade mesophyll			Spongy mesophyll			Palisade/Spongy mesophyll ratio			Xylem			Phloem			Stomatal density		
	Year																							
**Genotype**	**2018**	**2019**	**2020**	**2018**	**2019**	**2020**	**2018**	**2019**	**2020**	**2018**	**2019**	**2020**	**2018**	**2019**	**2020**	**2018**	**2019**	**2020**	**2018**	**2019**	**2020**	**2018**	**2019**	**2020**
1-3-2v	16.75	17.25	13.00	11.25	12.25	11.25	78.89	85.00	68.00	95.25	66.67	78.89	1.20	0.91	0.86	55.00	65.25	51.25	54.72	54.75	39.25	90	90	72
1-5-2v	19.25	15.50	12.50	13.00	11.67	11.00	74.25	95.00	73.25	106.75	76.00	85.50	0.98	0.91	0.87	56.94	47.50	44.50	50.83	45.00	39.00	93	87	80
1-6-2v	16.25	13.25	11.38	11.00	11.25	9.72	87.75	96.50	64.00	97.75	89.75	82.25	1.02	0.99	0.78	61.75	38.75	40.75	50.83	41.94	35.50	153	125	126
1-7-2v	18.89	17.25	15.71	13.50	11.00	11.00	85.31	100.50	76.00	94.25	75.75	76.75	1.18	1.07	1.02	40.56	34.50	35.50	37.00	32.75	30.25	135	120	121
1-10-2v	18.75	13.89	13.50	13.44	10.75	10.50	97.22	91.25	71.75	96.25	109.50	88.00	0.91	0.97	0.83	71.25	40.25	45.00	62.50	43.75	38.75	146	166	135
2-9-2v	18.25	14.75	12.22	15.25	12.75	11.75	104.50	100.75	75.25	109.50	106.25	80.00	0.98	0.92	0.96	40.83	40.00	35.28	45.75	35.00	30.28	150	122	136
2-10-2v	16.75	13.75	12.00	13.00	10.83	9.58	87.75	104.50	83.00	105.25	92.50	76.00	0.97	1.01	1.12	52.25	47.75	43.50	45.28	46.25	42.00	127	120	111
4-4-1v	12.75	12.75	9.88	10.75	11.25	10.75	79.06	84.50	57.36	97.50	75.28	78.61	1.02	0.87	0.74	35.75	37.78	37.75	32.50	36.50	29.75	95	79	76
4-7-1v	14.75	12.00	12.13	12.50	11.75	10.75	82.75	80.00	69.75	98.00	73.44	89.13	1.09	0.82	0.80	44.25	42.50	46.94	30.25	39.00	32.50	108	101	96
4-8-1v	13.61	12.75	13.25	10.50	10.25	11.38	80.75	92.00	73.44	90.25	82.50	77.25	1.00	1.03	1.00	40.75	33.44	37.50	41.50	37.75	33.25	110	93	97
5-3-1v	12.00	12.75	11.38	11.25	13.00	10.38	72.25	91.25	65.42	87.25	75.00	73.33	0.95	1.06	0.93	35.00	43.75	29.17	38.25	43.75	30.50	139	98	112
5-7-1v	15.00	13.25	12.75	11.25	11.25	9.72	87.00	99.25	62.08	78.93	87.00	58.93	1.03	1.31	1.02	42.25	42.25	39.75	38.75	44.00	36.25	144	136	121
1-1-2v	20.28	12.50	13.38	13.89	10.75	10.75	78.00	88.00	82.75	96.00	77.00	89.75	1.04	0.94	0.93	85.00	58.33	42.81	63.75	52.00	39.06	138	137	119
2-1-2v	17.75	13.33	12.75	13.25	11.50	11.00	71.25	85.28	71.75	95.75	93.50	91.25	0.78	0.91	0.79	51.50	38.61	46.50	44.25	37.25	35.25	113	93	95
2-2-2v	19.50	16.00	15.75	16.00	13.50	10.75	97.25	90.25	72.00	112.50	119.75	98.25	0.84	0.81	0.74	66.25	49.06	42.00	54.25	41.00	36.25	106	103	93
2-4-2v	19.17	13.50	15.13	14.25	10.56	10.75	77.50	91.75	67.50	93.25	70.75	74.75	1.07	1.00	0.92	47.00	41.00	31.50	30.50	37.25	24.50	108	91	101
2-8-2v	15.75	15.75	15.83	13.50	12.50	10.25	73.00	90.50	72.00	103.25	80.75	83.00	0.94	0.89	0.88	50.00	48.00	47.75	38.75	43.75	43.89	151	120	137
4-1-1v	16.00	13.75	12.50	13.00	9.50	11.13	67.75	76.50	62.00	89.00	67.19	74.25	1.13	0.95	0.93	42.00	37.25	35.75	30.50	39.00	34.17	154	139	123
4-2-1v	15.75	12.75	12.75	13.75	11.67	10.00	79.75	82.00	63.75	88.00	81.25	79.25	1.00	0.94	0.81	43.06	43.25	42.25	32.25	40.63	35.00	141	115	113
4-3-1v	15.25	14.00	13.00	12.50	12.00	11.13	66.75	69.50	56.50	78.75	74.75	71.50	0.90	0.90	0.82	33.33	33.75	32.25	30.00	30.50	24.00	138	119	133
4-9-1v	13.50	14.50	11.88	11.75	11.50	10.50	97.00	99.75	75.50	108.75	95.56	86.39	1.02	0.92	0.86	42.50	45.50	41.38	40.25	38.33	37.50	122	112	120
4-10-1v	13.75	12.50	13.06	10.50	11.00	11.13	84.25	87.50	70.00	90.83	76.75	80.00	0.98	0.87	0.84	35.75	50.00	43.63	33.50	38.50	32.50	106	90	84
5-1-1v	16.67	12.00	14.13	11.00	14.00	10.88	75.00	87.00	62.50	92.00	77.00	77.25	0.99	0.96	0.82	38.89	40.75	47.88	35.75	38.00	38.50	146	127	109
5-2-1v	13.50	12.00	11.13	10.00	9.75	8.75	71.25	85.25	76.75	80.50	72.75	71.50	1.00	1.06	1.10	39.50	37.75	41.75	37.78	34.50	36.00	114	84	80

5-4-1v	15.25	13.25	11.00	10.25	12.50	10.38	77.25	90.75	55.25	101.00	81.75	72.25	0.95	0.90	0.77	43.75	48.00	52.50	30.83	42.00	40.25	141	123	116
5-5-1v	16.25	15.25	13.38	10.75	11.50	9.25	71.25	93.75	72.50	89.75	82.75	74.25	0.88	1.05	0.98	41.75	42.25	39.75	21.00	46.50	35.75	109	87	98

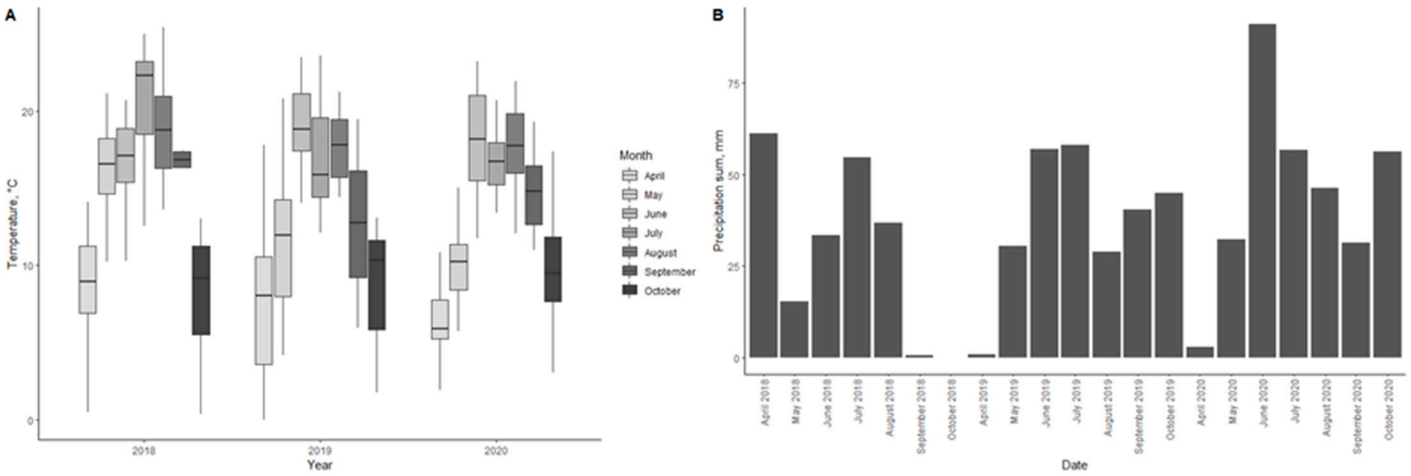

Figure S1. The temperature in the vegetation period throughout the three-year evaluation period (**A**) and the sum of precipitation (mm) during the three-year evaluation (**B**).

Analysis of vegetation season weather conditions during the three-year evaluation period

According to the data provided by the Latvian Environment, Geology and Meteorology Centre (LEGMC) [25], the spring of 2018 was characterized with heat records – the average temperature in Dobele was 1.5 °C above the seasonal norm, and precipitation was 17% below the norm. Summer had a heat record, with the temperature being 2.3 °C above the seasonal norm and precipitation being 39 % under the norm. Autumn was both hotter and drier than was the norm in all of Latvia, but there was little data from the weather station of Dobele, so a direct comparison cannot be made.

Similar weather abnormalities were recorded in 2019 – the temperature in spring was 1.6 °C above the seasonal norm, and precipitation was 46% below the norm. The summer of 2019 was slightly cooler than 2018, with the mean temperature being 1.4 °C above the norm and precipitation being 31 % below the norm. Autumn, however, was just as warm as the year before, with the temperature being 1.8 °C above the seasonal norm, but precipitation being 18 % under the seasonal norm.

In contrast, in 2020, the spring temperature was 0.1 °C above the norm, but the precipitation was 35 % below the norm. Heat records characterized the summer of 2020 in June and an overall seasonal temperature of 1 °C above the norm; precipitation was highest since 2017 but still 7 % below the norm. In contrast to the two previous years, autumn of 2020 was the warmest in the three-year study period, with the temperature being 3.2 °C above the norm, while precipitation was lower than in 2019 with 33 % below the norm.

When looking at precipitation data by month, the year 2018 was the year with the highest precipitation sum in April, whereas for July and September, the highest precipitation sum was in 2019. The months of June, August and October had the highest precipitation sum in 2020. However, it is important to note that there was a lack of precipitation data for September and October in 2018, but due to the overall trend of 2018 being a dry year, higher precipitation was reached in the following years.

The year 2018 stood out in terms of average temperature per month, as the mean temperature was highest for April, May, July, August and September. June and October had the highest mean temperature in 2019 and 2020, respectively.

Significant differences were found between the mean precipitation of April 2018 and April 2019 and April 2020 and May 2018 and 2019.

In the case of mean temperature values, significant differences were found between April 2018 and 2020, all three years in May, June 2018 and 2019 and between June 2018 and the following two years.
